# Clinical characteristics and prognosis analysis of acute symptomatic seizures secondary to autoimmune encephalitis

**DOI:** 10.3389/fneur.2024.1474888

**Published:** 2024-10-30

**Authors:** Mengyun Li, Qing Zhang, Xu Wang, Bofei Tan, Qiang Liu

**Affiliations:** ^1^First Clinical Medical College, Ningxia Medical University, Yinchuan, China; ^2^Department of Neurology, General Hospital of Ningxia Medical University, Yinchuan, China

**Keywords:** AE, seizure, clinical characteristics, prognosis, NLR

## Abstract

**Objective:**

This study aimed to analyze the clinical characteristics and prognosis of patients with autoimmune encephalitis (PWAE) who experienced seizures during the acute phase.

**Methods:**

Clinical data were collected from 84 patients diagnosed with AE at the General Hospital of Ningxia Medical University between January 2015 and January 2023. Patients were divided into seizure and non-seizure groups. Clinical characteristics of both groups were compared, including differences between anti-NMDAR and anti-LGI1 encephalitis within the seizure group. Due to the limited sample size and to avoid overfitting, we focused on univariate logistic regression analysis to identify individual prognostic factors.

**Results:**

A total of 84 patients were enrolled, with 76.19% (64/84) in the seizure group and 23.81% (20/84) in the non-seizure group. The seizure group had a longer hospital stay (*p* = 0.013), higher rates of impaired consciousness (*p* = 0.001), and more frequent intensive care unit (ICU) admission (*p* = 0.011). They also had higher peripheral blood neutrophil-to-lymphocyte ratio (NLR), leukocyte count, and uric acid levels (*p* = 0.038, *p* = 0.006, *p* = 0.020), and were more likely to show slow-wave rhythms on electroencephalography (EEG) (*p* = 0.031). At 2-year follow-up, there was no significant difference in prognosis between the seizure and non-seizure groups (*p* = 0.653), with 35.94% (23/64) of the seizure group having a poor prognosis. Status epilepticus (SE), complications, endotracheal intubation, mRS score at discharge, APE^2^, and RITE^2^ scores increased the risk of poor prognosis (OR > 1), while intensive care and albumin reduced the risk (OR < 1).

**Conclusion:**

Seizures are common in the early stages of AE, with faciobrachial dystonic seizures (FBDS) characteristic of anti-LGI1 encephalitis and SE and super-refractory status epilepticus (Sup-RSE) frequently observed in anti-NMDAR encephalitis. Seizure semiology across AE subtypes lacks specificity, and no symptoms clearly distinguish immune-mediated from non-immune causes. While seizures are linked to AE severity, particularly in anti-NMDAR encephalitis, they do not appear to impact overall prognosis. SE, complications, endotracheal intubation, modified Rankin Scale (mRS) score at discharge, Antibody-Prevalence in Epilepsy and Encephalopathy (APE^2^) score, Response to Immunotherapy in Epilepsy and Encephalopathy (RITE^2^) score, intensive care, and albumin were identified as significant prognostic factors.

## 1 Introduction

Seizures are a common and primary clinical manifestation in the early stages of AE, 42–100% of PWAE may develop seizures (1–3), with higher frequency in the acute phase, and it is the most common cause of new-onset refractory status epilepticus (NORSE), which can partially develop into Super-RSE (4). Most PWAE only show acute symptomatic seizures during the active phase of encephalitis, after adequate immunotherapy and encephalitis is controlled, the seizures are also controlled. However, a small proportion of PWAE (<15%) may develop chronic epilepsy (5). In 2020, the International League Against Epilepsy (ILAE) Autoimmunity and Inflammation Working Group (6) defined the former as “acute symptomatic seizures secondary to AE,” including seizures at the onset of AE and at the time of recurrence, and the latter is defined as “autoimmune epilepsy.” Compared with the neuronal surface antigen antibodies (which have direct functional effects, such as anti-NMDAR, GABA_B_R, LGI1, CASPR2, and AMPAR antibodies), neuronal intracellular antigen antibodies (typically mediated by cytotoxic T cells, such as Hu, CV2/CRMP5, Ma2, GAD65, and KLHL11 antibodies) mediate AE with a higher risk of developing autoimmune epilepsy.

AE is potentially clinically curable, with most patients having an overall good prognosis, though some may relapse. Prognosis varies by antibody subtype: AE associated with cell surface antigens tends to have better outcomes, while paraneoplastic AE targeting intracellular antigens has a poorer prognosis. Studies show that poor outcomes in anti-NMDAR encephalitis are linked to a Glasgow Coma Scale (GCS) score ≤ 8 at admission, cognitive impairment, positive serum antibodies, and delayed immunotherapy (7). Current research estimates the poor prognosis rate for anti-NMDAR encephalitis at 7.3–35.7% (8–11). For anti-LGI1 encephalitis, poor long-term outcomes are associated with ineffective initial treatment and relapse, with factors like advanced age and abnormal cerebrospinal fluid also contributing (12). The poor prognosis rate for anti-LGI1 encephalitis ranges from 4.70 to 51.20% (13–16).

Early immunotherapy can reduce seizure frequency or facilitate seizure termination in PWAE, and improve long-term outcomes (including cognitive function). However, the diagnosis of AE still relies on the detection of autoantibodies and requires clinicians to be sufficiently alert to AE; otherwise, immunotherapy may be delayed. Seizures are an important clinical manifestation in the early stages of AE and are suggestive of diagnosis. Rapid identification of seizures as autoimmune in origin can reduce the delay in immunotherapy caused by antibody detection. Therefore, a comprehensive understanding of the clinical characteristics of acute symptomatic seizures secondary to AE and the specificity of seizures in different AE subtypes can aid in the early diagnosis of AE and the identification of relevant neuro-specific antibodies.

In this study, we retrospectively collected and analyzed the clinical data (including demographic data, clinical manifestations, auxiliary tests, treatment regimens, and prognosis) of 84 PWAE admitted to the General Hospital of Ningxia Medical University. We analyzed the clinical characteristics of PWAE who developed acute symptomatic seizures, including those with anti-NMDAR, anti-LGI1, anti-CASPR2, anti-GABABR, anti-GAD65 encephalitis, and AE with the co-existence of multiple anti-neuronal antibodies. Additionally, we compared the clinical characteristics of anti-NMDAR encephalitis and anti-LGI1 encephalitis in those who developed acute symptomatic seizures. Furthermore, we examined the factors influencing the prognosis of PWAE who had seizures.

## 2 Materials and methods

### 2.1 Patients inclusion

A total of 84 PWAE who were admitted to the General Hospital of Ningxia Medical University from January 2015 to January 2023 were included in this study. The study was approved by the Ethics Committee of the General Hospital of Ningxia Medical University (Approval Number: KYLL-2024-1314) and has secured informed consent for the disclosure of information from all participants involved. All data in the study were thereby strictly anonymized. The inclusion criteria refer to the diagnostic criteria that proposed by Graus et al. (17) and the expert consensus on the diagnosis and treatment of AE in China (2022 edition) (18):

1. Acute or subacute onset, with one or more of the following symptoms:

- Limbic system symptoms: psychiatric symptoms, seizures, memory deficit.
- Encephalitis syndrome: clinical manifestations of diffuse or multifocal brain damage.
- Clinical manifestations of basal ganglia and/or diencephalon/hypothalamus involvement.
- Psychiatric disorders that do not qualify as non-organic diseases by a psychopsychologist.

1. at least one of the following:

- CSF lymphocytosis abnormality.
- Magnetic Resonance Imaging (MRI) suggests characteristic manifestation of AE.
- EEG suggests seizure or slow-wave activity.

1. Cell substrate-based assay (CBA) was positive for anti-NMDAR antibodies in CSF and serum; CBA-based detection of CSF and/or serum positive for other neuro-specific antibodies. Patients with positive antibodies only in serum need to have typical clinical symptoms and/or high antibody titers (> 1:32).
2. Other causes are reasonably excluded.

### 2.2 Exclusion criteria

Patients with a known history of epilepsy or those with seizures caused by other known conditions.

### 2.3 APE^2^ score and RITE^2^ score

To facilitate early identification of epilepsy related to AE, Dubey et al. (19, 20) introduced the APE^2^ score and the RITE^2^ score in 2018. The APE^2^ score consists of 10 criteria, with a maximum score of 18 points. A score of ≥4 suggests that epilepsy and encephalopathy are likely related to AE. The RITE^2^ score builds upon the APE^2^ score by adding two additional variables: (a) initiation of immunotherapy within 6 months of symptom onset; and (b) detection of membrane-specific autoantibodies. The RITE^2^ score has a total of 22 points, and a score of ≥7 indicates a positive response to immunotherapy. Both the APE^2^ and RITE^2^ scores have been shown to be effective tools for evaluating and managing autoimmune-associated epilepsy.

### 2.4 Analysis of clinical data

The demographic and clinical data of the patients, including age at onset, gender, total length of hospital stay, first symptoms, clinical manifestations, laboratory tests, MRI and EEG, treatments, and the modified mRS score at discharge were recorded. According to whether acute symptomatic seizures occurred during the acute phase of the disease, patients were divided into seizure group and non-seizure group, and the clinical data of the two groups were compared. Additionally, the clinical characteristics of patients with anti-LGI1 encephalitis and anti-NMDAR encephalitis in the seizure group were compared.

**Figure 1:**
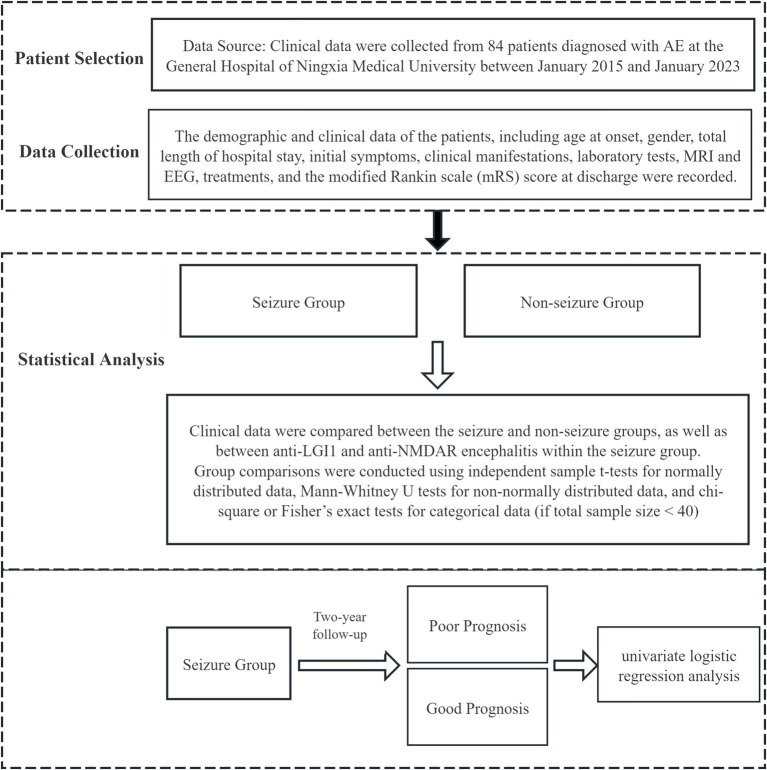
Flow diagram of the study process.

Patients in the seizure group were followed up through telephone or outpatient visits, and were classified based on the mRS score at the two-year follow-up. An mRS score ≥ two or death was defined as a poor prognosis, while an mRS score < two was defined as a good prognosis. Univariate logistic regression was conducted to identify predictors of prognosis in PWAE. The Study Process is shown in Figure 1.

### 2.5 Statistical methods

The study utilized SPSS 27 statistical software for analysis. Measurement data are presented as mean ± standard deviation for normally distributed variables or median (P25–P75) for non-normally distributed variables. An independent sample *t*-test was applied for comparisons between groups when the data followed a normal distribution. For non-normally distributed data, the Mann–Whitney U test was conducted. Categorical data are expressed as frequencies or percentages, and comparisons between groups were made using the chi-square test, or Fisher’s exact test when the total sample size was less than 40. Univariate logistic regression was conducted to identify predictors of prognosis in PWAE. Given the limited sample size, our multivariate analyses showed indications of overfitting, which could affect the accuracy of the model’s predictions. As a result, we opted to focus on the univariate logistic regression analysis.

## 3 Results

### 3.1 Grouping and antibody distribution

**Figure 2:**
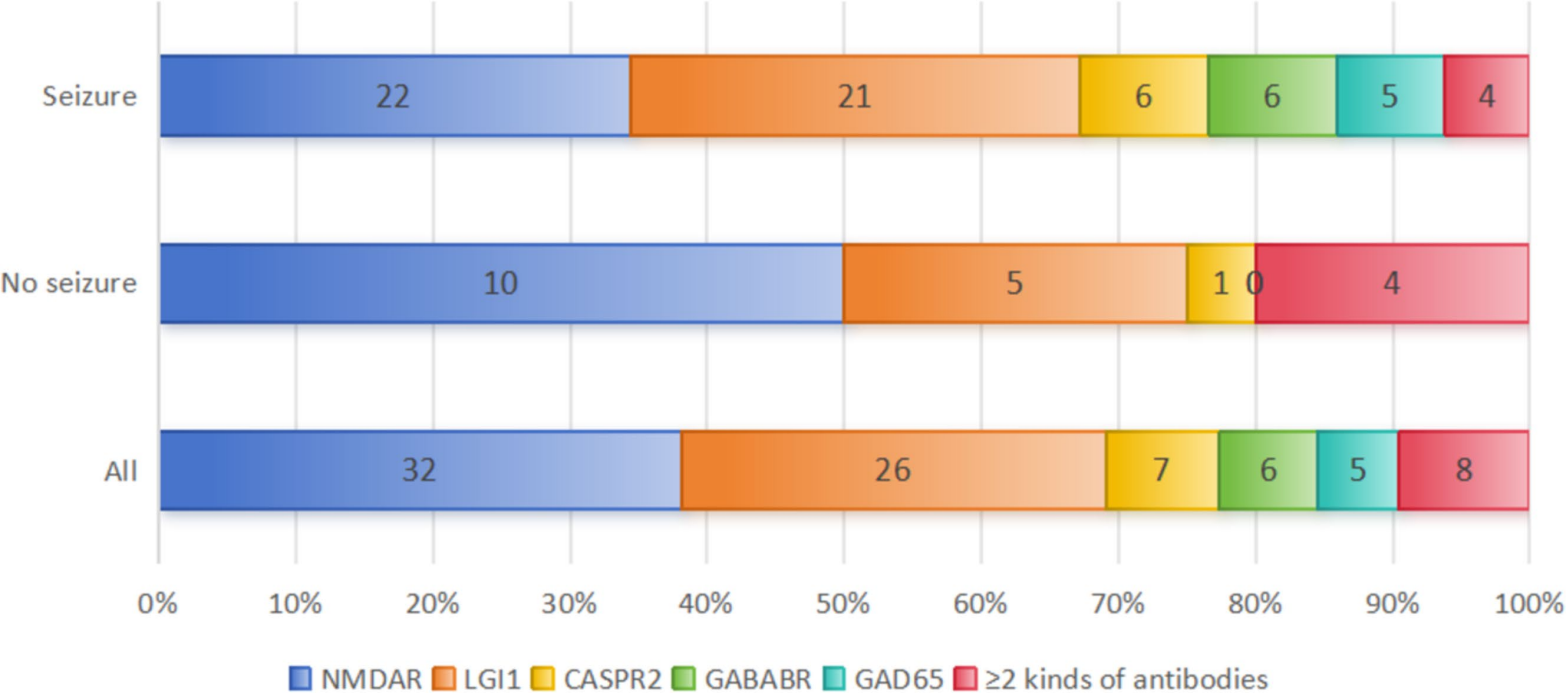
Distribution of AE antibody types between the two groups. A total of 8 patients with co-existence of multiple anti-neuronal antibodies were included in this study: 4 patients in the seizure group (anti-NMDAR and mGluR5 antibody positive; anti-LGI 1 and GFAP antibody positive; anti-AMPAR1, AMPAR2 and anti-SOX 1 antibodies positive; anti-GABA_B_R, anti-GAD 65, -SOX 1 and Ma2 positive). And 4 patients in the non-seizure group (anti GlyR 1 and GFAP antibodies positive; anti-NMDAR and MOG antibodies positive; anti-LGI 1 and CASPR2 antibodies positive; anti-mGluR5 and MOG antibodies positive).

This study included a total of 84 PWAE. Among them, 76.19% (64/84) were in the seizure group, and 23.81% (20/84) were in the non-seizure group. The antibody distribution in the two groups is shown in Figure 2, and there was no significant difference in antibody distribution between the two groups (Table 1). Among PWAE who experienced seizures, 56.25% (36/64) had seizures as the initial symptom. Of these, 54.68% (35/64) experienced only one type of seizure, while 45.31% (29/64) had two or more types. Integrating the seizure symptomatology with the EEG findings, we observed that focal seizures were identified in 64.06% (41/64), generalized seizures in 25.00% (16/64), and mixed seizures in 10.94% (7/64). Additionally, 51.56% (33/64) progressed to SE, with 10.94% (7/64) advancing to Sup-RSE. All PWAE who progressed to Sup-RSE with anti-NMDAR antibody positive, six were young females (three with concomitant ovarian teratoma), and one was a young male.

**Table 1:** Demographic data and clinical data of two groups.

Antibodies	Seizure group (*n* = 64)	Non-seizure group (*n* = 20)	*p*-value
Demographic data			
Gender (male/female)	38/26	11/9	0.729
Age (years), median (IQR)	43.50(29.00 ~ 57.75)	50.00 (32.00 ~ 61.50)	0.440
Hospital days, median (IQR)	18.00 (11.00 ~ 27.75)	11.50 (9.00 ~ 15.50)	0.013*
Intensive care n /(%)	26 (40.63)	2 (10.00)	0.011*
Antibodies			
NMDAR n/(%)	22 (34.38)	10 (50.00)	0.198
LGI1 n/(%)	21 (32.81)	5 (25.00)	
GABA_B_R n/(%)	6 (9.38)	0	
CASPR2 n/(%)	6 (9.38)	1 (5.00)	
GAD65 n/(%)	5 (7.81)	0	
Multiple anti-neuronal antibodies n /(%)	4 (6.25)	4 (20.00)	
Clinical manifestation			
Prodromal symptoms n /(%)	18 (28.13)	8 (40.00)	0.316
Psychiatric and behavioral abnormalities n (%)	40 (62.50)	14 (70.00)	0.541
Cognitive impairment, n /(%)	32 (50.00)	11 (55.00)	0.696
Involuntary movement n /(%)	26 (40.63)	5 (25.00)	0.206
Autonomic dysfunction n /(%)	22 (34.38)	5 (25.00)	0.433
Consciousness disturbance n /(%)	42 (65.63)	5 (25.00)	0.001*
Tumor, n /(%)	4 (6.25)	0	0.568
Blood routine, liver and kidney function, and electrolytes			
Serum potassium (mmol/l), median (IQR)	3.98 (3.70 ~ 4.30)	3.90 (3.55 ~ 4.11)	0.182
Serum sodium (mmol/l), median (IQR)	140.15 (134.25 ~ 142.15)	138.05 (134.95 ~ 142.55)	0.805
Serum chloride ion (mmol/l), median (IQR)	103.90 (97.85 ~ 107.08)	106.10 (98.83 ~ 107.90)	0.339
Leukocyte count (*10^9/l), median (IQR)	9.05 (7.08–11.24)	6.34 (5.57–8.36)	0.006*
NLR, median (IQR)	4.26 (2.52 ~ 6.17)	2.62 (1.73 ~ 4.49)	0.038*
Albumin (g/l), median (IQR)	41.95 (36.60 ~ 45.28)	42.00 (36.70 ~ 44.69)	0.777
Uric acid (μmol/l), median (IQR)	293.00 (202.50 ~ 391.75)	211.50 (145.25 ~ 297.75)	0.020*
Initial lumbar puncture results			
Intracranial pressure (mmH_2_O), Median (IQR)	170.00 (127.50 ~ 200.00)	150.00 (107.50 ~ 187.50)	0.181
Elevated CSF leukocytes n (%)	30 (46.87)	10 (50.00)	0.807
Elevated CSF neutrophils n (%)	18 (28.13)	6 (30.00)	0.871
Elevated CSF plasma cells n (%)	9 (14.06)	1 (5.00)	0.275
CSF-TP (g/l), median (IQR)	0.46 (0.34 ~ 0.67)	0.48 (0.37 ~ 0.80)	0.546
CSF-Glu (mmol/l), median (IQR)	3.40 (3.00 ~ 4.05)	3.35 (2.90 ~ 3.90)	0.664
CSF-CI (mmol/l), median (IQR)	121.00 (118.00 ~ 124.00)	123.00 (117.00 ~ 126.00)	0.729
CSF-IgG (mg/l), median (IQR)	37.40 (23.15 ~ 55.50)	41.15 (23.60 ~ 154.73)	0.414
Cranial MRI abnormalities n (%)	37/62 (59.68)	10/19 (52.63)	0.586
Unilateral/Bilateral	14/23	2/8	0.457
Involving temporal lobe and hippocampus n (%)	29/37 (78.38)	10/10 (100.00)	0.028*
EEG abnormalities n (%)	49/59 (83.05)	13/19 (68.42)	0.170
Slow-wave rhythm (Yes/No)	29/30	4/15	0.031*
Prognosis			
mRS score at discharge	2.92 ± 1.44	2.50 ± 1.36	0.250
Good functional outcome (mRS ≤2) n/%	38 (59.38)	13 (65.00)	0.653
Poor functional outcome (mRS > 2) n/%	26 (40.63)	7 (35.00)	

Asterisks (*) indicate statistically significant differences (*P* < 0.05).

Among the included PWAE, anti-NMDAR encephalitis accounted for the highest proportion at 38.10% (32/84), followed by anti-LGI1 encephalitis at 30.95% (26/84), AE with co-existence of multiple anti-neuronal antibodies at 9.52% (8/84), anti-CASPR2 encephalitis at 8.33% (7/84), anti-GABA_B_R encephalitis at 7.14% (6/84), and anti-GAD65 encephalitis at 5.95% (5/84). The different AE subtypes have different pathogenesis, and their seizure occurrence rates also vary. In our study, seizures occurred in 68.75% (22/32) of anti-NMDAR encephalitis, 80.77% (21/26) of anti-LGI1 encephalitis, 85.71% (6/7) of anti-CASPR2 encephalitis, 100.00% (6/6) of anti-GABA_B_R encephalitis, 100.00% (5/5) of anti-GAD65 encephalitis, and 50.00% (4/8) of AE with co-existence of multiple anti-neuronal antibodies patients during their disease course (Figure 2). We conducted a comparative analysis of the data of anti-NMDAR encephalitis and anti-LGI1 encephalitis with seizures. Due to the small number of cases for other AE subtypes, we only performed data statistics.

### 3.2 General demographic data and clinical manifestations

The median age of the 84 PWAE was 46.00 (29.00 ~ 58.75) years, with males accounting for 58.33% (49/84) and females accounting for 41.67% (35/84). The median age of the seizure group was 43.50 (29.00 ~ 57.75) years, lower than that of the non-seizure group at 50.00 (32.00 ~ 61.50) years. Both groups were predominantly male, with no significant differences in age and gender between the two groups (*p >* 0.05) (Table 1).

The main clinical manifestations of PWAE included psychiatric and behavioral abnormalities, seizures, cognitive impairment, Autonomic Dysfunction and involuntary movements. Severe cases could present with consciousness disturbances and central hypoventilation. Some PWAE showed prodromal symptoms such as fever, headache, nausea, vomiting, diarrhea, and flu-like symptoms within 2 weeks before the onset. In this study, there were no significant differences in the incidence of prodromal symptoms, psychiatric and behavioral abnormalities, cognitive impairment, Autonomic dysfunction and involuntary movements between the two groups (*p >* 0.05). Consciousness disturbances occurred in 55.95% (47/84) of PWAE, with significantly more occurrences in the seizure group compared to the non-seizure group [65.63% (42/64) vs. 25.00% (5/20), *p* = 0.001]. Due to the severity of the illness, 33.33% (28/84) of patients required intensive care treatment, with a significantly higher proportion in the seizure group compared to the non-seizure group [40.63% (26/64) vs. 10.00% (2/20), *p* = 0.011]. The median hospital stay for the 84 PWAE was 16.00 (10.00 ~ 26.50) days, with a significantly longer stay for the seizure group compared to the non-seizure group [18.00 (11.00 ~ 27.75) vs. 11.50 (9.00 ~ 15.50), *p* = 0.013] (Table 1).

Among the AE subtypes with seizures, anti-NMDAR encephalitis was more common in the young, with an equal number of males and females affected, while anti-LGI1 encephalitis was more common in elderly males. There were significant differences in the gender distribution and median age between these two subtypes (*p* = 0.076, *p* < 0.001). In 90.91% (20/22) of patients with anti-NMDAR encephalitis, consciousness disturbances were observed; 59.09% (13/22) required intensive care treatment; 77.27% (17/22) progressed to SE; and 31.82% (7/22) progressed to Sup-RSE, all of which showed significant differences compared to LGI1 encephalitis (*p* = 0.005, *p* = 0.004, *p* < 0.001, *p* = 0.021). We also found that anti-NMDAR encephalitis was the only AE subtype resulted in Sup-RSE, with a significant difference in seizure type compared to LGI1 encephalitis (*p* = 0.021), and its median hospital stay was the longest among all AE subtypes, significantly longer than that of LGI1 encephalitis [27.50 (16.75 ~ 46.50) vs. 11.00 (7.00 ~ 18.00), *p* < 0.001]. However, there was no significant difference in the classification of seizures between the two AE subtypes (*P*<0.05), with focal seizures being predominant in both. Anti-LGI1 encephalitis is commonly associated with temporal lobe onset seizures. In our study, 23.81% (5/21) of patients with anti-LGI1 encephalitis experienced prodromal symptoms such as piloerection, loss of consciousness, visual abnormalities, upper abdominal discomfort, and sensory disturbances before seizures; 14.29%(3/21) exhibited FBDS. Moreover, anti-LGI1 encephalitis had the highest proportion of cognitive impairment, with a significant difference compared to anti-NMDAR encephalitis [71.43% (15/21) vs. 31.82% (7/22), *p* = 0.009]. Notably, among all AE subtypes, anti-GAD65 encephalitis had the highest female incidence rate at 80.00% (4/5), and anti-NMDAR encephalitis had the lowest median onset age at [27.50 (18.75 ~ 32.25)] years (Table 2).

**Table 2:** Clinical data of different AE subtypes in the seizure group.

Variable	Antibody						*P*-value
	NMDAR (*n* = 22)	LGI1 (*n* = 21)	GABA_B_R (*n* = 6)	CASPR2 (*n* = 6)	GAD65 (*n* = 5)	Multiple anti-neuronal antibodies (*n* = 4)	NMDAR/LGI1
Demographic data							
Male/Female	11/11	16/5	4/2	4/2	1/4	2/2	0.076*
Age (years), Median (IQR)	27.50 (18.75 ~ 32.25)	55.00 (48.00 ~ 68.50)	57.00 (46.00 ~ 63.53)	54.00 (40.75 ~ 61.00)	54.00 (40.75 ~ 61.00)	50.00 (27.00 ~ 62.50)	<0.001*
Hospitalization days (days), median (IQR)	27.50 (16.75 ~ 46.50)	11.00 (7.00 ~ 18.00)	25.00 (16.00 ~ 40.00)	20.50 (12.50 ~ 39.00)	16.00 (8.50 ~ 22.50)	20.00 (11.00 ~ 65.00)	<0.001*
Intensive care n /(%)	13 (59.09)	3 (14.29)	4 (66.67)	3 (50.00)	0	3 (75.00)	0.004*
Onset with seizuren n/(%)	9 (40.91)	13 (61.90)	4 (66.67)	4 (66.67)	4 (80.00)	2 (50.00)	0.169
Classification of seizures							
Generalized seizures	6 (27.27)	3 (14.29)	2 (33.33)	2 (33.33)	1 (20.00)	2 (50.00)	0.568
Focal seizures	12 (54.55)	15 (71.43)	4 (66.67)	4 (66.67)	4 (80.00)	2 (50.00)	
Mixed seizures	4 (18.18)	3 (14.29)	0	0	0	0	
SE n/(%)	17 (77.27)	5 (23.81)	5 (83.33)	2 (33.33)	1 (20.00)	3 (75.00)	<0.001*
Super-RSE n/(%)	7 (31.82)	0	0	0	0	0	0.021*
Prodromal symptoms n /(%)	8 (36.36)	6 (28.57)	2 (33.33)	1 (16.67)	1 (20.00)	0	0.586
Psychiatric and behavioral abnormalities n (%)	14 (63.64)	16 (76.19)	3 (50.00)	2 (33.33)	1 (20.00)	4 (100.00)	0.370
Cognitive impairment n /(%)	7 (31.82)	15 (71.43)	4 (66.67)	2 (33.33)	2 (40.00)	2 (50.00)	0.009*
Involuntary movement n /(%)	14 (63.64)	8 (38.10)	1 (16.67)	1 (16.67)	1 (20.00)	1 (25.00)	0.094
Autonomic dysfunction n /(%)	6 (27.27)	6 (28.57)	3 (50.00)	4 (66.67)	2 (40.00)	1 (25.00)	0.199
Tumor n /(%)	2 (9.10)	0	0	0	0	2 (50.00)	0.488
Blood routine, liver and kidney function, and electrolytes							
Serum sotassium (mmol/l), Mean ± SD	3.98 (3.70 ~ 4.24)	4.00 (3.73 ~ 4.37)	4.05 (3.47 ~ 4.50)	3.91 (3.64 ~ 4.23)	4.04 (3.23 ~ 4.46)	4.01 (3.49 ~ 4.30)	0.846
Serum sodium ions (mmol/l), Median (IQR)	142.40 (140.30 ~ 146.45)	136.80 (129.05 ~ 140.50)	136.45 (133.30 ~ 140.08)	140.85 (134.90 ~ 143.45)	140.70 (136.95 ~ 144.25)	131.80 (129.85 ~ 144.40)	<0.001*
Serum chloride (mmol/l), median (IQR)	104.20 (101.33 ~ 107.55)	99.50 (96.65 ~ 104.20)	102.40 (96.33 ~ 108.33)	107.90 (102.93 ~ 110.88)	140.70 (136.95 ~ 144.25)	98.15 (97.40 ~ 103.48)	0.019*
Leukocyte count (*10^9/l), Median (IQR)	10.15 (8.27 ~ 11.90)	8.52 (6.76 ~ 9.62)	8.23 (6.14 ~ 12.96)	13.40 (7.39 ~ 21.83)	5.73 (4.79 ~ 8.80)	8.91 (7.47 ~ 11.40)	0.017*
NLR, median (IQR)	5.09 (2.68 ~ 6.43)	2.95 (2.30 ~ 4.60)	4.84 (3.55 ~ 12.17)	8.51 (4.28 ~ 14.95)	1.97 (0.92 ~ 3.69)	4.94 (4.10 ~ 7.32)	0.017*
Albumin (g/l), median (IQR)	44.10 (40.45 ~ 46.45)	41.80 (37.45 ~ 45.97)	38.98 (30.79 ~ 44.51)	39.50 (34.30 ~ 44.83)	42.40 (32.74 ~ 44.78)	35.60 (27.55 ~ 46.13)	0.319
Uric acid (μmol/l), Median (IQR)	308.00 (233.00 ~ 444.75)	273.00 (221.50 ~ 363.50)	299.00 (170.75 ~ 374.25)	318.00 (217.50 ~ 658.75)	215.00 (173.00 ~ 412.50)	151.00 (64.50 ~ 415.25)	0.296
Initial lumbar puncture results							
Intracranial pressure (mmH_2_O), Mean ± SD	211.52 ± 49.91	139.58 ± 53.73	157.50 ± 46.88	160.83 ± 41.28	127.50 ± 15.55	188.75 ± 16.52	<0.001*
Elevated CSF leukocytes n (%)	19 (86.36)	3 (14.29)	3 (50.00)	2 (33.3)	1 (20)	2 (50)	<0.001*
Elevated CSF neutrophils n (%)	13 (59.09)	1 (4.76)	1 (16.67)	2 (33.3)	0	1 (25)	<0.001*
Elevated CSF plasma cells n (%)	5 (22.73)	1 (4.76)	1 (16.67)	0	0	2 (50)	0.185
CSF-TP (g/l), Median (IQR)	0.38 (0.28 ~ 0.54)	0.51 (0.40 ~ 0.70)	0.51 (0.29 ~ 1.21)	0.72 (0.42 ~ 1.14)	0.47 (0.34 ~ 0.55)	0.61 (0.42 ~ 3.32)	0.04*
CSF-Glu (mmol/l), Median (IQR)	3.05 (2.68 ~ 3.95)	3.60 (3.10 ~ 4.35)	3.64 (2.55 ~ 4.43)	3.70 (2.78 ~ 4.38)	3.20 (3.15 ~ 4.75)	3.20 (0.98 ~ 6.05)	0.086
CSF-CI (mmol/l), median (IQR)	123.00 (119.75 ~ 125.45)	118.00 (115.50 ~ 121.50)	122.50 (118.50 ~ 126.25)	121.50 (117.00 ~ 125.75)	124.00 (119.50 ~ 125.00)	118.50 (116.25 ~ 124.50)	0.003*
Cranial MRI abnormalities n (%)	6/20 (30.00)	15/21 (71.43)	6/6 (100.00)	4/6 (66.67)	3/5 (60.00)	3/4 (75.00)	0.008*
Unilateral/Bilateral	3/3	5/10	3/3	2/2	0/3	1/2	–
Involving temporal lobe and hippocampus n (%)	4/6 (66.67)	13/15 (86.67)	5/6 (83.33)	1/4 (25.00)	3/3 (100.00)	3/3 (100.00)	–
EEG abnormalities n (%)	20/22 (90.90)	13/19 (68.42)	6/6 (100.00)	4/4 (100.00)	4/5 (80.00)	2/3 (66.67)	0.115
Slow-wave rhythms n (%)	15/20 (75.00)	7/13 (53.85)	2/6 (33.33)	2/4 (50.00)	2/4 (50.00)	1/2 (50.00)	0.270
Prognosis							
APE^2^ score, mean ± SD	8.00 ± 2.18	6.24 ± 2.23	7.00 ± 3.03	5.67 ± 1.63	5.20 ± 1.64	8.00 ± 3.83	0.012*
RITE^2^ score, mean ± SD	11.91 ± 2.10	10.05 ± 2.46	11.00 ± 3.03	9.33 ± 2.16	8.80 ± 2.17	12.00 ± 3.83	0.011*
Good functional outcome (mRS ≤ 2) n/%	16 (72.73)	12 (57.14)	4 (66.67)	2 (33.33)	2 (40.00)	2 (50.00)	0.284
Poor functional outcome (mRS > 2) n/%	6 (27.27)	9 (42.86)	2 (33.33)	4 (66.67)	3 (60.00)	2 (50.00)	

Asterisks (*) indicate statistically significant differences (*P* < 0.05).

Tumors are potential triggers for AE. In this study, 73 PWAE underwent tumor marker testing, with 35.62% (26/73) showing abnormal results. Further screening of these patients revealed four cases with tumors: three young females had ovarian teratomas (two were anti-NMDAR positive; one was positive for both anti-NMDAR and mGluR5), and one 45-year-old male had small cell lung cancer (positive for anti-GABA_B_R, GAD65, SOX1, and Ma2). All of these patients experienced seizures. Additionally, One 61-year-old male with anti-GABA_B_R encephalitis and significantly elevated CA12-5 levels had a chest CT suggestive of lung cancer, but no further pathological examination was conducted (the patient refused).

### 3.3 Auxiliary examinations

#### 3.3.1 Blood routine, liver and kidney function, and electrolytes

All PWAE underwent blood routine, liver and kidney function tests, and electrolyte assays upon admission. The peripheral blood leukocyte count, NLR, and uric acid levels were significantly higher in the seizure group compared to the non-seizure group (*p* = 0.006, *p* = 0.038, *p* = 0.020) (Table 1). Within the seizure group, the leukocyte count and NLR were significantly higher in patients with anti-NMDAR encephalitis compared to those with anti-LGI1 encephalitis (*p* = 0.017), but there was no significant difference in uric acid levels between the two groups. Patients with anti-CASPR2 encephalitis had the highest peripheral blood leukocyte count, NLR, and uric acid levels among the six AE subtypes. Hyponatremia is a common complication of LGI1 encephalitis. In our study, the median serum sodium and chloride levels were significantly lower in patients with anti-LGI1 encephalitis compared to those with anti-NMDAR encephalitis (*p* < 0.001, *p* = 0.019). However, patients with the co-existence of multiple anti-neuronal antibodies had the lowest median serum sodium and chloride levels among all AE subtypes (Table 2).

#### 3.3.2 Initial lumbar puncture results

All PWAE underwent lumbar puncture (one patient completed this at an external hospital). Intracranial pressure was measured in 75 patients (58 in the seizure group and 17 in the non-seizure group), and CSF-IgG was measured in 57 patients (41 in the seizure group and 16 in the non-seizure group). There were no significant differences in the median values of intracranial pressure, CSF leukocytes, CSF-TP, CSF-glu, CSF-CI, and CSF-IgG between the two groups (*p >* 0.05) (Table 1). In the seizure group, the average intracranial pressure, CSF leukocyte count, and CSF neutrophil elevation rate in anti-NMDAR encephalitis were significantly higher than those in LGI1 encephalitis (*p* < 0.001), and these were the highest among all subtypes (Table 2).

#### 3.3.3 Cranial magnetic resonance imaging and electroencephalography

A total of 81 PWAE underwent MRI, with 58.02% (47/81) showing unilateral or bilateral T2 or FLAIR signal abnormalities. Among PWAE with abnormal MRI signals, the lesions primarily involved the temporal lobe and/or hippocampus [82.98% (39/47)]. This included 78.38% (29/37) in the seizure group and 100.00% (10/10) in the non-seizure group, with a significant difference between the two groups (*p* = 0.028) (Table 1). In the seizure group, anti-LGI1 encephalitis was more likely to show abnormal MRI signals compared to anti-NMDAR encephalitis (*p* = 0.008) (Table 2).

EEG in PWAE often exhibited diffuse or multifocal slow-wave rhythms or focal epilepsy or epileptiform discharges (located in the temporal lobe or extratemporal areas). In this study, 78 PWAE underwent EEG, and 79.49% (62/78) showed abnormal EEG rhythms. Among them, 53.23% (33/62) displayed slow-wave rhythms. PWAE in the seizure group had significantly more slow-wave EEG patterns compared to the non-seizure group (*p* = 0.049) (Table 1).

### 3.4 Prognosis

A poor prognosis was defined as an mRS at the 2-year follow-up ≥ 2 or death, while a good prognosis was defined as an mRS at the 2-year follow-up < 2. The prognosis and mRS score at discharge with no significant difference observed between the seizure and non-seizure groups (*p* = 0.653, *p* = 0.250) (Table 1).

APE^2^ score can identify seizures of autoimmune origin, and the RITE^2^ score can predict the response to immunotherapy (19, 20). We assessed the APE^2^ and RITE^2^ scores of 64 PWAE in the seizure group to evaluate their relationship with prognosis. As shown in Table 3, we conducted univariate logistic regression analysis to evaluate the impact of each potential influencing factor on the prognosis of PWAE with acute symptomatic seizures. The results indicated that SE (*p* = 0.009, OR = 4.427, 95% CI: 1.441–13.602), number of complications (*p* = 0.030, OR = 1.279, 95% CI: 1.024–1.597), endotracheal intubation (*p* = 0.009, OR = 4.500, 95% CI: 1.461–13.863), mRS score at discharge (*p* = 0.001, OR = 2.027, 95% CI: 1.315–3.122), APE^2^ score (*p* = 0.027, OR = 1.294, 95% CI: 1.030–1.625), and RITE^2^ score (*p* = 0.015, OR = 1.325, 95% CI: 1.055–1.664) increased the risk of poor prognosis, while intensive care (*p* = 0.016, OR = 0.266, 95% CI: 0.091–0.779) and albumin (*p* = 0.038, OR = 0.911, 95% CI: 0.834–0.995) reduced the risk of poor prognosis (OR < 1) (Table 3).

**Table 3:** Univariate logistic regression analysis of prognosis in the seizure group.

Variable	OR	95%CI	*P*-value
Gender	0.629	0.223–1.773	0.381
Age	0.028	0.998–1.059	0.065
Hospital days	1.022	0.996–1.050	0.096
Intensive care	0.266	0.091–0.779	0.016*
Clinical manifestation			
Prodromal symptoms	1.057	0.347–3.222	0.922
Types of seizures	0.730	0.343–1.551	0.413
Classification of seizures			
Generalized seizures	3.840	0.422–34.936	0.232
Mixed seizures	3.600	0.345–37.616	0.285
Focal seizures (constant)	0.167	–	0.097
SE	4.427	1.441	0.009*
Psychiatric and behavioral abnormalities	1.619	0.548–4.786	0.384
Cognitive impairment	1.145	0.412–3.183	0.795
Involuntary movement	1.589	0.564–4.477	0.381
Autonomic dysfunctionn	3.810	0.326–44.495	0.286
Number of complications	1.279	1.024–1.597	0.030*
Consciousness disturbance	2.550	0.792–8.212	0.117
Endotracheal intubation	4.500	1.461–13.863	0.009*
Initial lumbar puncture results			
Intracranial pressure	1.001	0.992–1.009	0.815
CSF leukocytes	1.003	0.994–1.012	0.503
CSF neutrophils	0.942	0.851–1.043	0.248
CSF lymphocyte	1.005	0.993–1.017	0.394
CSF monocyte	1.021	0.924–1.128	0.687
CSF-TP	0.545	0.164–1.806	0.320
CSF-Glu	1.226	0.813–1.848	0.331
CSF-CI	1.026	0.932–1.134	0.581
CSF-IGg	1.002	0.982–1.021	0.830
Blood routine, liver and kidney function, and electrolytes			
Serum sotassium	0.986	0.316–3.079	0.981
Serum sodiumions	0.999	0.929–1.074	0.970
Serum chloride	0.976	0.898–1.062	0.572
Leukocyte	1.005	0.885–1.140	0.943
NLR	0.024	0.938–1.117	0.595
Albumin	0.911	0.834–0.995	0.038*
Uric acid	1.000	0.997–1.002	0.875
mRS score at discharge	2.027	1.315–3.122	0.001*
Cranial MRI abnormalities			
Unilateral/Bilateral	1.157	0.292–4.587	0.835
EEG abnormalities			
Slow-wave rhythm	1.053	0.358–3.086	0.926
APE^2^ score	1.294	1.030–1.625	0.027*
RITE^2^ score	1.325	1.055–1.664	0.015*

Asterisks (*) indicate statistically significant differences (*P* < 0.05).

## 4 Discussion

Seizures are a common and primary clinical manifestation in the early stages of PWAE. They are characterized by a high incidence, diverse and complex seizure types, and resistance to medication. However, acute symptomatic seizures secondary to AE are potentially curable and typically resolve completely once central nervous system (CNS) inflammation is controlled. Only a minority of cases may progress to chronic epilepsy. Studies (21) have shown that PWAE mediated by antibodies against intracellular antigens have a higher risk of developing epilepsy compared to those with AE associated with antibodies against neuronal cell surface antigens. This may be related to the direct neuromodulatory effects of surface antigen-targeting autoantibodies through antigen modulation, immune cell recruitment, or complement activation. These types of AE respond well to immunotherapy and often achieve seizure-free status (22). In this retrospective study, we collected clinical data from 84 PWAE, including cases of anti-NMDAR, anti-LGI1, anti-CASPR2, anti-GABA_B_R, anti-GAD65 antibody encephalitis, and AE with co-existence of multiple anti-neuronal antibodies. We focused on analyzing the clinical characteristics of patients with acute symptomatic seizures secondary to AE and compared the clinical characteristics of the most common subtypes, anti-NMDAR and anti-LGI1 encephalitis.

In our study, 76.19% (64/84) of PWAE experienced seizures, which is consistent with the findings of de Bruijn et al. (23), with focal seizures being the predominant type. Among these, anti-NMDAR encephalitis has the highest proportion (38.10%, 32/84), followed by LGI1 encephalitis (30.95%, 26/84), AE with co-existence of multiple anti-neuronal antibodies was the least common subtype, accounting for 9.52% (8/84) of cases. Anti-NMDAR encephalitis is the most common AE subtype, with approximately 75% of cases experiencing seizures (24). Seizures typically occur in the early stage of the disease course, with no significant gender difference, and predominant seizure types are complex partial seizures (CPS) and generalized tonic–clonic seizures (GTCS). This group has the highest incidence of NORSE, which may be associated with ovarian teratomas (25). In our study, 68.75% (22/32) of patients with anti-NMDAR encephalitis experienced seizures, which Consistent with previous studies, showing an equal incidence between males and females. In 40.91% (9/22) of these patients, seizures were the initial symptom. The seizures in anti-NMDAR encephalitis patients were diverse, including focal, generalized, and mixed seizures, and predominantly consisting of focal seizures. At the same time, it is the AE subtype with the highest proportion of mixed seizures and accounted for all cases of Sup-RSE (31.82%, 7/22), including 2 cases associated with ovarian teratomas, consistent with previous studies.

Previous studies have reported that 80.8% of patients with anti-LGI1 encephalitis may experience seizures (26), commonly in middle-aged and elderly males. These patients often have temporal lobe seizures associated with autonomic dysfunction and cognitive impairment and may be accompanied by bilateral dystonic-like seizures, sensory disturbances, confusion, and altered consciousness. In our study, 80.77% (21/26) of patients with anti-LGI1 encephalitis experienced seizures, consistent with previous research findings. Focal seizures were predominant, primarily manifesting as simple partial seizures (SPS), CPS, GTCS, or combinations of these types (with or without autonomic dysfunction). Prodromal symptoms such as piloerection, loss of consciousness, visual abnormalities, upper abdominal discomfort, and sensory disturbances were observed in 23.81% (5/21) of these patients. Cognitive impairment was present in 71.43% (15/21) of cases, the highest proportion among all AE subtypes, aligning with previous studies. FBDS are characteristic of anti-LGI1 encephalitis, observed in 14.29% (3/21) of our cases, lower than the 28–69% reported in other studies (27–29). This discrepancy may be due to recall bias in our retrospective study and a lack of awareness among early clinical practitioners regarding these symptoms.

Anti-GAD65 antibodies are among the most frequently detected antibodies in adult patients with chronic refractory epilepsy. GAD is an intracellular enzyme that influences the metabolism of the excitatory neurotransmitter glutamate, catalyzing its conversion to GABA, the primary inhibitory neurotransmitter in the CNS. The humoral immune response against anti-GAD65 antibodies reduces the conversion of glutamate to GABA, increasing the excitatory neurotransmitter glutamate and thus enhancing seizure susceptibility. In our study, all 5 patients with anti-GAD65 encephalitis developed acute symptomatic seizures, with 80.00% (4/5) being female. During follow-up (> 6 months), 4 patients continued to experience seizures of varying degrees and required long-term oral anti-seizure medications (ASMs).

CASPR2 is a transmembrane protein and a common binding site for VGKC antibodies. Seizures associated with CASPR2 encephalitis may result from disrupted interaction between CASPR2 and contactin-2, impairing Kv1 channel expression and leading to hyperexcitability and network dysfunction. Our study included 7 patients with anti-CASPR2 encephalitis, 6 of whom experienced seizures, with a higher incidence in males, consistent with other studies (30, 31) in Asia.

Anti-GABA_B_R encephalitis commonly affects middle-aged and elderly males and is characterized by severe and refractory seizures in the early stage of the disease course. Seizures may be the only symptom, and indeed, seizures have become a hallmark of anti-GABA_B_R encephalitis, necessitating consideration in cases of new-onset refractory epilepsy in middle-aged and elderly patients. Our cohort included 6 patients with anti-GABA_B_R encephalitis, all of whom experienced significant seizures in the early stage of disease course, typically of more than one seizure type, predominantly GTCS. SE occurred in 83.33% (5/6) of these patients, the highest proportion among AE subtypes.

Seizures can significantly impact the severity of AE, particularly in anti-NMDAR encephalitis. In our study, PWAE who experienced seizures had longer median hospital stays, higher proportions of consciousness disturbances, and a greater need for intensive care compared to those without seizures. However, after receiving adequate immunotherapy and ASMs, the prognosis between the two groups showed no significant difference. Among PWAE who experienced seizures, those with anti-NMDAR encephalitis had the longest hospital stays and the highest proportions of consciousness disturbances and need for intensive care, which is related to its progression to SE and Sup-RSE. In our study, 77.27% (17/22) of patients with anti-NMDAR encephalitis developed SE, and 31.82% (7/22) developed Sup-RSE, making it the only AE subtype to present with Sup-RSE. Notably, the incidence of SE in our patients with anti-NMDAR encephalitis was higher than in other studies (27). Jeannin-Mayer et al. (32) suggested in their study on EEG analysis of anti-NMDAR encephalitis that abnormal EEG findings, such as rhythmic delta activity, movement disorders, and impaired consciousness, could often be mistaken for SE, possibly explaining our higher incidence compared to other studies.

Tumors are a potential trigger for AE, and different antibody types of AE are associated with different tumors. Anti-NMDAR encephalitis is commonly associated with ovarian teratomas, likely due to the presence of neural tissue in the teratomas. Chefdeville et al. (33) found that the presence of neural tissue was significantly higher in teratomas associated with anti-NMDAR encephalitis compared to controls (*p* < 0.001). Dalmau et al. (34, 35) reported that up to 58% of young women with anti-NMDAR encephalitis had ovarian teratomas, higher than the 14.3–47.8% reported in Asian studies (18). In our study, three PWAE had ovarian teratomas, all of whom were women of childbearing age. Two of these patients were positive for anti-NMDAR antibodies and developed Sup-RSE during their disease course; they had good prognoses after tumor resection. One patient was positive for both anti-NMDAR and anti-mGluR5 antibodies, and her family refused surgical treatment. She died from gastrointestinal hemorrhage after AE recurrence.

Anti-GABA_B_R encephalitis is often associated with small cell lung cancer, though the mechanism remains unclear. Lancaster et al. (36) found that 47% of patients with anti-GABA_B_R encephalitis had small cell lung cancer, most commonly in middle-aged and elderly males. In our cohort, one 61-year-old male with anti-GABA_B_R encephalitis had a large, irregular nodule in the left upper lung on chest CT, with highly suspicious malignant cells found in the exfoliative cytology, suggesting lung cancer. However, his family refused further pathological examination, and he was only treated with intravenous immunoglobulin (IVIg) and ASMs, dying 2 years after discharge. Another patient, a 42-year-old male positive for anti-GABA_B_R, GAD65, Ma2, and SOX1 antibodies, was diagnosed with small cell lung cancer and treated with IVIg, ASMs, and platinum-based chemotherapy, dying 1 year after diagnosis. Anti-LGI1, anti-CASPR2, and anti-GAD65 encephalitis are rarely associated with tumors. However, some cases of anti-LGI1 and anti-CASPR2 encephalitis may be associated with thymomas (37). Anti-GAD65 antibodies can be found in some patients with lung cancer (small cell and non-small cell), breast cancer, thymoma, and testicular seminoma (38–40). Therefore, continuous and regular malignancy screening is recommended for patients with anti-GAD65 encephalitis. In our study, we did not find any such patients.

NLR is a newly discovered marker reflecting immune responses to various infectious and non-infectious stimuli, representing both innate immunity (neutrophils) and adaptive immunity (lymphocytes). Innate immune responses are rapid but short-lived, whereas adaptive immune responses are slower but long-lasting. The normal range of NLR is 1.0–2.0, with pathological values being >3.0 or < 0.7 in adults, and values between 2.3 and 3.0 may serve as an early warning of pathological states (41, 42). Studies have indicated that peripheral blood NLR has diagnostic value for AE and can be a reliable biomarker for assessing AE severity and prognosis. Zhang et al. (43) found that NLR is an independent risk factor for moderate to severe status in the initial phase of anti-NMDAR encephalitis, with a critical value >4.232. Seizures are also associated with systemic inflammatory changes reflected in peripheral leukocyte counts and NLR (44). Higher NLR (within 24 h of seizure onset) is associated with SE and can independently predict SE occurrence. In our study, PWAE who experienced seizures had significantly higher NLR levels and peripheral blood leukocyte counts compared to those without seizures (*p* < 0.05), suggesting that NLR may predict seizure occurrence in PWAE. We compared NLR between anti-NMDAR and anti-LGI1 encephalitis and found that NLR and leukocyte counts were significantly higher in patients with anti-NMDAR encephalitis (*p* < 0.05). Among all subtypes, anti-CASPR2 encephalitis had the highest NLR and leukocyte counts. However, compared to other AE subtypes, the incidence of SE in patients with anti-CASPR2 encephalitis was relatively low (33.33%), which contradicts the role of NLR as a predictor of SE. Apart from anti-CASPR2 encephalitis, other subtypes such as anti-NMDAR, anti-LGI1, anti-GAD65, and anti-GABA_B_R encephalitis exhibited NLR values that were largely consistent with SE incidence, suggesting that the predictive value of NLR may vary across different AE subtypes. This discrepancy could be due to the unique pathophysiological mechanisms of anti-CASPR2 encephalitis, potentially related to specific inflammatory pathways or other clinical factors such as complications or treatment protocols. The current study’s sample size may also be a limiting factor, and future studies with larger sample sizes are needed to further explore the applicability of NLR as a predictor of SE across different AE subtypes. Additionally, we found that PWAE who experienced seizures had higher uric acid levels compared to those without seizures (*p* < 0.05). Uric acid is the final metabolic product of purines. Beamer et al. (45) discovered that blood purine levels are effective biomarkers for seizures and epilepsy, with acute seizures and epilepsy which are associated with increased purine levels.

EEG is currently the most objective method for diagnosing seizures and determining seizure types. It plays a crucial role in diagnosing and evaluating the effectiveness of treatment for acute symptomatic seizures secondary to AE. A large retrospective study (24) involving 3,722 PWAE found that 70% of patients experienced seizures during their disease course, with 85% showing abnormal EEG results. The most common type of EEG abnormality was slow-wave changes (51.10%), consistent with our findings. In our study, 76.19% (64/84) of PWAE experienced seizures, and 79.49% (62/78) had abnormal EEG rhythms, primarily diffuse or multifocal slow-wave changes. Baysal-Kirac et al. (46) conducted a comparative study of PWAE who experienced seizures, comparing those who were positive for specific serum antibodies to those who were negative. They found that serum-positive patients’ EEGs were more likely to show nonconvulsive status epilepticus, diffuse rhythmic delta waves, frontal intermittent rhythmic delta activity (FIRDA), and periodic lateralized epileptiform discharges (PLEDs) in the temporal lobe. Delta brushes, characterized by superimposed 20–30 Hz beta waves on 1–3 Hz delta waves, are considered a distinctive EEG pattern in adult patients with anti-NMDAR encephalitis and are associated with AE severity and prognosis. Patients with delta brush patterns usually have a poorer prognosis. In our study, two patients with anti-NMDAR encephalitis had delta brush patterns on EEG, both of whom initially presented with psychiatric and behavioral abnormalities, followed by seizures that quickly progressed to SE, requiring intensive care.

In this study, we analyzed the prognostic factors of PWAE who developed acute symptomatic seizures. Our results revealed no significant difference in the mRS score at discharge and prognosis between the seizure and non-seizure groups (*p* > 0.05), suggesting that acute symptomatic seizures may not significantly impact the overall prognosis of AE patients. We used univariate logistic regression analysis to assess the impact of each potential influencing factor on the prognosis of patients with acute symptomatic seizures secondary to AE. The results indicated that SE, the number of complications, endotracheal intubation, mRS score at discharge, APE^2^ score, and RITE^2^ score increased the risk of poor prognosis (OR > 1), while intensive care and albumin reduced the risk of poor prognosis (OR < 1). While our analysis showed that intensive care was associated with a better prognosis, it is important to clarify that intensive care itself is often provided to patients with more severe conditions, such as SE, multiple complications, or those requiring endotracheal intubation. These factors are typically linked to poor outcomes. However, in our study, patients with SE receiving intensive care had better outcomes compared to those who did not (59.09%[13/22] vs. 36.36%[11/22]). This suggests that, despite the severity of their condition, ICU interventions may help improve prognosis by providing more intensive care and monitoring. Due to the limited sample size, we observed signs of overfitting in our multivariate analyses, which could compromise the reliability of the model’s predictions. As a result, we decided to focus on univariate logistic regression analysis. Although univariate analysis does not account for potential confounding factors, it provides valuable insights into the individual effects of specific variables. Future studies with larger sample sizes will be necessary to confirm these findings and to conduct a more robust multivariate analysis that can better account for confounding variables and interactions.

It is important to acknowledge the limitations of this study. First, the small sample size may limit the generalizability of the findings, particularly for certain antibody subtypes such as anti-CASPR2, anti-GABA_B_R, and anti-GAD. Due to the limited number of patients in these subgroups, we only provided descriptive analysis without conducting statistical comparisons, as the small sample sizes could lead to unreliable or non-significant results. Additionally, due to concerns about overfitting, we focused on univariate analysis. Second, recall bias may have affected the accuracy of clinical history, as some data relied on patient or family recollection. To minimize this, we primarily used hospital records and clinical follow-ups documented by healthcare professionals, which are relatively objective and reliable. Third, the single-center design may introduce selection bias, as the patient population may not reflect broader demographics. Future studies should aim to increase the sample size and involve multiple centers to improve representativeness.

In summary, seizures are a common and primary clinical manifestation in the early stages of AE. FBDS is characteristic of LGI1 encephalitis, while SE and Sup-RSE are frequently observed in NMDAR encephalitis. Beyond these, the seizure semiology of different AE subtypes lacks specificity, and no other seizure symptoms reliably differentiate immune-mediated causes from non-immune etiologies. Seizures in AE are associated with disease severity, particularly in anti-NMDAR encephalitis, although the presence of acute symptomatic seizures does not significantly impact the overall prognosis of PWAE. Univariate analysis identified SE, complications, endotracheal intubation, mRS score at discharge, APE^2^ score, RITE^2^ score, intensive care, and albumin as significant prognostic factors. These findings suggest that early identification and active intervention, especially in AE subtypes prone to seizures, can improve prognosis. Tailored treatment strategies based on antibody profiles may further enhance patient outcomes. Long-term follow-up is essential to monitor the potential development of chronic epilepsy, and future prospective studies are needed to confirm these findings and assess the impact of early intervention on long-term outcomes.

## Data Availability

The raw data supporting the conclusions of this article will be made available by the authors, without undue reservation.
